# Antitumor and Antimicrobial Potential of Bromoditerpenes Isolated from the Red Alga, *Sphaerococcus coronopifolius*

**DOI:** 10.3390/md13020713

**Published:** 2015-01-26

**Authors:** Daniel Rodrigues, Celso Alves, André Horta, Susete Pinteus, Joana Silva, Gérald Culioli, Olivier P. Thomas, Rui Pedrosa

**Affiliations:** 1 Marine Resources Research Group (GIRM), ESTM, Polytechnic Institute of Leiria, 2520-641 Peniche, Portugal; E-Mails: daniel.rodrigues@imbe.fr (D.R.); celso.alves@ipleiria.pt (C.A.); andre.horta@ipleiria.pt (A.H.); susete.pinteus@ipleiria.pt (S.P.); joana.m.silva@ipleiria.pt (J.S.); 2 Nice Institute of Chemistry-PCRE, UMR 7272 CNRS, University de Nice-Sophia Antipolis, Parc Valrose, 06108 Nice, France; E-Mails: olivier.thomas@unice.fr (O.P.T.); culioli@univ-tln.fr (G.C.); 3 MAPIEM, EA 4323, Université de Toulon, 83957 La Garde, France; 4 Mediterranean Institute of Biodiversity and Ecology, Marine and Continental, Rue de la Batterie des Lions, 13007 Marseille, France; 5 Centre of Pharmacology and Chemical Biopathology, Faculty of Medicine, University of Porto, 4200-319 Porto, Portugal

**Keywords:** red algae, *Sphaerococcus*, diterpenes, sphaerane, dactylomelane, HepG-2, pathogenic microorganisms

## Abstract

Cancer and infectious diseases continue to be a major public health problem, and new drugs are necessary. As marine organisms are well known to provide a wide range of original compounds, the aim of this study was to investigate the bioactivity of the main constituents of the cosmopolitan red alga, *Sphaerococcus coronopifolius*. The structure of several bromoditerpenes was determined by extensive spectroscopic analysis and comparison with literature data. Five molecules were isolated and characterized which include a new brominated diterpene belonging to the rare dactylomelane family and named sphaerodactylomelol (**1**), along with four already known sphaerane bromoditerpenes (**2**–**5**). Antitumor activity was assessed by cytotoxicity and anti-proliferative assays on an *in vitro* model of human hepatocellular carcinoma (HepG-2 cells). Antimicrobial activity was evaluated against four pathogenic microorganisms: *Escherichia coli*, *Pseudomonas aeruginosa*, *Staphylococcus aureus* and *Candida albicans*. Compound **4** exhibited the highest antimicrobial activity against *S. aureus* (IC_50_ 6.35 µM) and compound **5** the highest anti-proliferative activity on HepG-2 cells (IC_50_ 42.9 µM). The new diterpene, sphaerodactylomelol (**1**), induced inhibition of cell proliferation (IC_50_ 280 µM) and cytotoxicity (IC_50_ 720 µM) on HepG-2 cells and showed antimicrobial activity against *S. aureus* (IC_50_ 96.3 µM).

## 1. Introduction

Cancer and infectious diseases continue to be one of the major public health concerns, and consequently, there is a perpetual need for new chemotherapeutics to fight new diseases and drug resistance. In the last few decades, nature has played a significant role as a source of new drugs, and recent trends in drug research emphasize that the marine environment has a high potential for the discovery of new pharmaceuticals [1,2,3]. Marine ecosystems are among the richest and most complex ones in terms of biodiversity. Original chemical and physical conditions in such an environment provide conditions for the production of quite specific and potent active molecules. Among other reasons, marine organisms have been found to produce original and bioactive substances because they are living in an exigent, competitive and aggressive environment [4,5]. These characteristics render marine organisms ideal candidates as novel sources of both preexisting and unrecognized high value-added biomolecules with potential for providing sustainable economic and human benefits [6]. Marine algae have been one of the richest and promising sources of bioactive specialized metabolites that probably have diverse simultaneous functions for their producer and can act, for example, as antimicrobial, antifouling and herbivore deterrents or as ultraviolet-screening agents [7,8,9]. These defensive strategies can result in a high level of structural and chemical diversity for the metabolites, originating from different metabolic pathways with great pharmaceutical and biomedical potential [10,11]. Marine algae-originated compounds have been found to be associated with numerous health-promoting effects, including, in particular, anti-oxidative, anti-inflammatory, antiviral, antimicrobial or anticancer effects [10,12]. Well-documented bioactive metabolites of marine algae include mainly brominated phenols, polysaccharides, but especially, a large variety of terpenoids, several of them being halogenated [13,14,15,16].

Since its first chemical analysis in 1976, the cosmopolitan red alga, *Sphaerococcus coronopifolius*, has yielded a large number of interesting brominated cyclic diterpenes, most of them containing one or two bromine atoms [17,18,19,20,21,22,23,24,25,26,27,28,29,30,31,32,33,34,35,36,37,38,39,40]. Concerning the biological activities of these compounds, some of them have already demonstrated antibacterial activity against Gram-positive bacteria [33], and others have been assayed for their cytotoxicity against human lung cancer cell lines [37] or their antibacterial activity against multidrug-resistant and methicillin-resistant *Staphylococcus aureus* strains [38,39]. Several bromoditerpenes have also been screened against the model organism, *Amphibalanus amphitrite*, in order to evaluate their antifouling properties.

The aim of the present study was to address the antitumor and antimicrobial bioactivity characterization of the major brominated diterpenes isolated from samples of *S. coronopifolius* collected in the Atlantic, while most of the chemically-studied specimens of this algal species were collected from the Mediterranean or the Adriatic seas.

## 2. Results and Discussion

### 2.1. In Vitro Bioactivity-Guided Fractionation

The screening of antitumor and antimicrobial activities of compounds isolated from *S. coronopifolius* was performed for the methanol (MeOH) and dichloromethane (CH_2_Cl_2_) extracts of this alga. The CH_2_Cl_2_ extract exhibited the highest anti-proliferative and antimicrobial activities (Table 1 and Table 2, respectively). Consequently, in order to isolate and identify the compounds responsible for these biological activities, this extract was further studied. It was first fractionated by normal phase vacuum liquid chromatography (VLC) on silica with eluents of increasing polarities (from cyclohexane to EtOAc), yielding five fractions (F1–F5 through a 25% step). In the first bioassay, fraction F2 exhibited the strongest cytotoxicity (IC_50_ 104 µg/mL) and anti-proliferative (19.8 µg/mL) activity (Table 1). On the other hand, fractions F1, F3, F4 and F5 demonstrated much more potency in the inhibition of HepG-2 cell proliferation than in the reduction of their viability. In antimicrobial assays (Table 2), the highest growth inhibition was measured against *S. aureus* for fractions F2 and F3 with IC_50_ of 5.10 and 5.39 µg/mL, respectively. In the antifungal assay, the highest activity was exhibited against *C. albicans* by fraction F2 with an IC_50_ of 53.9 µg/mL.

**Table 1:** Cytotoxicity and anti-proliferative (IC_50_) effects induced on HepG-2 cells by crude extracts and VLC fractions of *S.* *coronopifolius*. IC_50_ values are expressed as the means of eight independent experiments with 95% confidence intervals.

		IC_50_ (µg/mL)		
		Cytotoxicity	Anti-Proliferative	
**Crude extracts**	MeOH	470.6 (310.7–712.6)	646.5 (398.4–1049.0)	
	CH_2_Cl_2_	14.13 (8.12–24.60)	32.32 (22.37–46.70)	
**VLC fractions**	F1	>1000	102.5 (68.08–154.2)	
	F2	104.3 (81.82–132.9)	19.78 (13.79–28.38)	
	F3	>1000	70.17 (38.78–127.0)	
	F4	>1000	36.68 (23.37–57.55)	
	F5	>1000	39.32 (25.89–59.71)	

According to these results, several VLC fractions showed interesting effects against different targets, and it was decided to purify and structurally characterize the main components of fractions F2 and F3, due to their high anti-proliferative and antimicrobial effects, respectively.

**Table 2:** Antimicrobial activities (IC_50_) against *E. coli*, *P. aeruginosa*, *S. aureus* and *C. albicans* of crude extracts and VLC fractions obtained from *S. coronopifolius*. IC_50_ values are expressed as the means of eight independent experiments with 95% confidence intervals.

		IC_50_ (µg/mL)			
		*E*. *coli*	*P*. *aeruginosa*	*S*. *aureus*	*C*. *albicans*
**Crude Extracts**	MeOH	>1000	>1000	73.65 (58.52–92.69)	>1000
	CH_2_Cl_2_	267.1 (231.5–308.1)	363.1 (207.0–637.1)	25.15 (13.47–46.96)	435.9 (285.6–665.5)
**VLC Fractions**	F1	107.0 (92.02–124.5)	338.7 (248.3–461.9)	16.49 (10.19–26.66)	78.61 (58.12–106.3)
	F2	228.4 (188.7–276.4)	141.5 (117.6–170.4)	5.10 (4.50–5.78)	538.9 (308.4–941.6)
	F3	>1000	599.9 (307.4–1171.0)	5.39 (4.19–6.93)	>1000
	F4	433.9 (358.5–525.1)	436.4 (184.1–1035.0)	6.45 (5.16–8.05)	>1000
	F5	757.0 (614.5–932.6)	422.8 (291.9–612.4)	13.16 (9.98–17.35)	>1000

### 2.2. Isolation and Structure Elucidation of the Major Compounds of Fractions F2 and F3 

**Figure 1:**
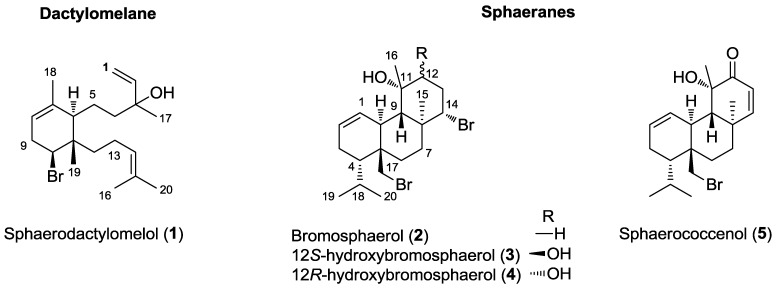
Chemical structure of the five bromoditerpenes, **1**–**5**, isolated from *S. coronopifolius.*

The HPLC purifications of the selected VLC fractions, F2 and F3, followed by the structural identification of the major compounds by NMR and MS led to the identification of five diterpenes belonging to two distinct families: one new dactylomelane (**1**) and four known sphaeranes (**2**–**5**) (Figure 1). It was not surprising to find compounds belonging to the sphaerane class of diterpenes in our samples. In comparison with literature data, we were thus able to identify, unambiguously, bromosphaerol (**2**) [31], 12*S*-hydroxybromosphaerol (**3**) [34], 12*R*-hydroxybromosphaerol (**4**) [34] and sphaerococcenol (**5**) [31] as additional major metabolites of the CH_2_Cl_2_ extract of *S. coronopifolius* harvested on the Portuguese coast.

Compound **1** was isolated as an optically-active oil, and its (+)-HRESIMS spectrum (see Supplementary Information) revealed an isotopic pattern characteristic of the presence of one bromine atom at *m/z* 351.17117 and 353.16891 (intensities 1:1) that was consistent with the molecular formula C_20_H_32_Br^+^. The ^1^H NMR spectrum (see Supplementary Information) confirmed the presence of a diterpene with five characteristic methyl signals at δ_H_ 0.89 (s, H_3_-19), 1.30 (s, H_3_-17), 1.63 (s, H_3_-16), 1.69 (s, H_3_-20) and 1.70 (s, H_3_-18), the chemical shifts of the last three revealing three methyls substituted on olefinic double bonds (Table 3). A terminal monosubstituted double bond was evidenced by the vinylic signals at δ_H_ 5.92 (dd, *J* = 17.2 and 10.8 Hz, H-2), 5.22 (dd, *J* = 17.2 and 1.2 Hz, H-1a) and 5.09 (dd, *J* = 10.8 and 1.2 Hz, H-1b). Two additional trisubstituted double bonds were inferred from the NMR signals at δ_H_ 5.22–5.17 (m, H-8), δ_C_ 120.5 (CH, C-8) and 137.2 (qC, C-7) and at δ_H_ 5.12–5.08 (m, H-14), δ_C_ 124.3 (CH, C-14) and 131.8 (qC, C-15).

**Table 3:** ^1^H (500 MHz) and ^13^C (125 MHz) NMR data of sphaerodactylomelol (**1**).

Atom n°	δ_H_ in ppm, mult. (*J* in Hz)	δ_C_ in ppm, mult.
1	5.09, dd (10.8, 1.2) 5.22, dd (17.2, 1.2)	112.3, CH_2_
2	5.92, dd (17.2, 10.8)	144.9, CH
3	-	73.6, qC
4	1.83, td (13.1, 4.7) 1.46 m	44.2, CH_2_
5	1.63, tdd (13.1, 4.7, 2.0) 1.38–1.29, m	23.3, CH_2_
6	2.11–2.06, m	45.0, CH
7	-	137.2, qC
8	5.22–5.17, m	120.5, CH
9	2.62–2.49, m	35.3, CH_2_
10	4.31, dd (10.2, 6.2)	61.5, CH
11	-	41.4, qC
12	1.57, ddd (14.4, 12.4, 5.1) 1.43 m	38.6, CH_2_
13	2.03, dd (12.9, 5.8) 1.90–18.1, m	21.5, CH_2_
14	5.12–5.08, m	124.3, CH
15	-	131.8, qC
16	1.63, s	17.9, CH_3_
17	1.30, s	27.9, CH_3_
18	1.70, s	22.4, CH_3_
19	0.89, s	16.8, CH_3_
20	1.69, s	25.9, CH_3_

**Figure 2:**
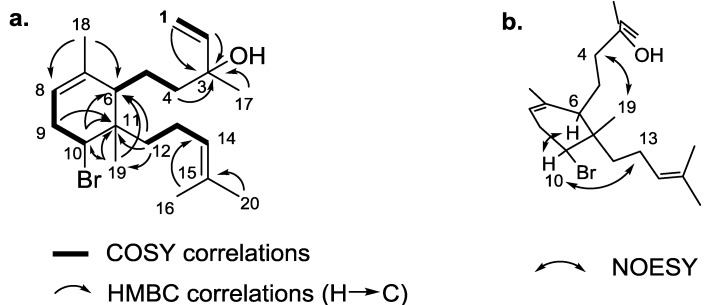
Key COSY, HMBC (**a**) and NOESY (**b**) correlations for sphaerodactylomelol (**1**).

It appeared that the terminal double bond was connected to a quaternary carbon, as evidenced by the absence of further scalar coupling from the vinylic protons. The H-1 and H-2/C-3 HMBC correlations indicated that the chemical shift of the quaternary carbon was δ_C_ 73.6 (qC, C-3), which was characteristic of an allylic alcohol (Figure 2a). The conclusion on the presence of an alcohol and not a bromine atom at this position was also given by the observation of a second heteroatom-substituted carbon at δ_H_ 4.31 (dd, *J* = 10.2 and 6.2 Hz, H-10), δ_C_ 61.5 (CH, C-10), with chemical shifts fully consistent with a brominated methine. Consequently, the isotopic pattern obtained by HRESIMS was the result of dehydration of the allylic and tertiary alcohol at C-3 [M − H_2_O + H]^+^, and the molecular formula of **1** was then determined to be C_20_H_33_BrO. In view of the NMR data, from the four unsaturations corresponding to this molecular formula, the last one was assigned to a cycle. As described in Figure 2a, full analysis of ^1^H-^1^H COSY and HMBC spectra (see Supplementary Information) allowed the establishment of the carbon skeleton of compound **1**. More precisely, the closing of the precursor geranylgeranyl chain through the C-6/C-11 bond was ascertained by the key H_3_-19, H-10, H_2_-12/C-6 and H_3_-19, H-10, H-9/C-11 HMBC correlations, thus leading to the six-membered ring. The key H_2_-12/C-6, C-11, C-19 HMBC correlations placed the last H-12/H-13/H-14 spin coupled system at C-11, thus revealing the full planar structure of **1**. We relied on the NOESY spectrum (see Supplementary Information) for the elucidation of the relative configuration of **1**. H-10/H-6 nOe placed the bromine on the same side as the linear alkyl chain at C-6, while the second alkyl chain was placed on the other side, thanks to H_3_-18/H_2_-4 and H-10/H_2_-13 nOes (Figure 2b). The absolute configuration was not determined, due to the lack of simple assessment. However, we assume the same stereochemistry as the one determined for the known analogues, **2**, **3** and **4**.

This compound does not belong to the usual sphaerane family found in this species, but it is another rare example of the dactylomelane family (Figure 1) [41]. To date, there are nine compounds of this class of diterpenes described in the literature. Eight of them are characterized by a bridged oxide between C-7 and C-10, thus leading to a 7-oxabicyclo[2.2.1]heptane moiety. These compounds were isolated from sea hares of the genus *Aplysia*, but also from a red alga of the genus *Laurencia*, thus confirming the diet of the sea hare [41,42,43]. The last compound of this chemical class was isolated from the same species, *S. coronopifolius*, harvested also along the Atlantic coast [33]. Its chemical structure is very similar to that of compound **1**, differing by the presence of an allylic alcohol at C-14 instead of a dimethyl-substituted double bond. Interestingly, all of the compounds isolated from samples of *S. coronopifolius* collected in the Mediterranean and Adriatic seas belong to the sphaerane family, suggesting that the presence of dactylomelanes would be restricted to Atlantic specimens.

### 2.3. Antitumor and Antimicrobial Activities of Purified Compounds 

The results of the anti-proliferative experiments demonstrated that all isolated compounds have anti-proliferative activity on HepG-2 cells at sub-toxic concentrations (Table 4). The highest inhibition of cell proliferation was exhibited by compound **5** and then compound **4** with IC_50_ of 42.9 and 105 μM, respectively. Sphaerococcenol A (**5**) has already demonstrated high cytotoxic activities against A549, OE21, PC-3 and LoVo cell lines, with LC_50_ of 3.7, 3.0, 3.7 and 2.8 µM, respectively [37]. However, this is the first report of activity against HepG-2 cells. In the same report, 12*R*-hydroxybromosphaerol (**4**) also showed cytotoxic activity against the four cell lines with LC_50_ of 28, 25, 26 and 26 μM, respectively. The absence of cytotoxicity was verified in the case of NSCLC-N6 (L16) and A549 cell lines [34]. On the other hand, the major reduction of HepG-2 cell viability was induced by the new compound **1** with an IC_50_ of 720 µM, which also showed an anti-proliferative activity (IC_50_ 280 µM).

**Table 4:** Cytotoxicity and anti-proliferative effects (IC_50_) induced on HepG-2 cells by bromoditerpenes **1**–**5** isolated from *S. coronopifolius* and drugs (positive controls). IC_50_ values are expressed as the means of eight independent experiments with 95% confidence intervals.

		IC_50_ (µM)		
		Cytotoxicity	Anti-Proliferative	
**Bromoditerpenes**	**1**	719.85 (519.79–996.81)	279.93 (206.78–378.74)	
	**2**	>1000	203.33 (90.65–456.18)	
	**3**	>1000	291.42 (206.22–411.83)	
	**4**	>1000	104.83 (55.27–198.89)	
	**5**	>1000	42.87 (22.76–78.88)	
**Drugs (+)**	Cisplatin	454.6 (388.9–531.3)	75.41 (61.78–92.05)	
	Tamoxifen	>1000	45.68 (31.84–65.57)	

Regarding antimicrobial activity (Table 5), the highest growth inhibition against *S. aureus* was obtained for compounds **4** (IC_50_ 6.35 µM), **2** (IC_50_ 22.4 µM) and **1** (IC_50_ 96.3 µM). This is the first report for an antimicrobial activity of 12*R*-hydroxybromosphaerol (**4**). For sphaerococcenol A (**5**), a previous report showed an antimalarial activity against the chloroquine-resistant *Plasmodium falciparum* FCB1 strains with an IC_50_ of 1 µM, but no results has been published on antimicrobial activities [33].

## 3. Experimental Section 

### 3.1. General Experimental Procedures

UV and CD spectra were measured using a Jasco J-810 spectropolarimeter. Optical rotation was measured on a Perkin-Elmer 343 polarimeter, using a 100-mm microcell. IR spectra were recorded on a Bruker Tensor 27 spectrophotometer. NMR spectra were measured on a Bruker Avance 500-MHz spectrometer. Chemical shift values (δ) were reported in parts per million (ppm) relative to the appropriate internal solvent standard, and coupling constants (*J*-values) are given in Hertz and referenced to residual solvent signals (CDCl_3_ at δ_H_ 7.26 and δ_C_ 77.16). HPLC purifications were carried out on a Jasco LC-2000 system equipped with a PU-2087 Plus preparative pumping system and a UV-2075 Plus detector. HRESIMS data were conducted on an LTQ Orbitrap mass spectrometer (Thermo Fisher Scientific, Waltham, MA, USA).

**Table 5:** Antimicrobial activities (IC_50_) against *E. coli*, *P. aeruginosa*, *S. aureus* and *C. albicans* of bromoditerpenes **1**–**5** isolated from *S. coronopifolius* and drugs (positive controls). IC_50_ values are expressed as the means of eight independent experiments with 95% confidence intervals.

		IC_50_ (µM)			
		*E*. *coli*	*P*. *aeruginosa*	*S*. *aureus*	*C*. *albicans*
**Bromoditerpenes**	**1**	>100	>100	96.30 (84.60–109.61)	>100
	**2**	>100	>100	22.42 (15.44–32.57)	>100
	**3**	>100	>100	>100	>100
	**4**	>100	>100	6.35 (4.78–8.42)	>100
	**5**	>100	>100	>100	>100
**Drugs (+)**	Ampicillin	6.42 (1.86–22.26)	-	0.11 (0.08–0.15)	-
	Bacitracin	>100	-	2.85 (2.36–3.44)	-
	Chloramphenicol	>100	-	80.49 (58.99–109.86)	-
	Oxytetracycline	1.12 (0.65–1.89)	2.13 (1.65–2.76)	0.87 (0.59–1.32)	-
	Amphotericin b	-	-	-	>100
	Flumequine	-	-	-	>100

### 3.2. Sampling, Identification and Treatment of Algal Material

*Sphaerococcus coronopifolius* samples were collected freshly from Berlenga Nature Reserve (39°24'44.8"N 9°30'29.5"W), Peniche (Portugal), in June 2012, and immediately transported to the laboratory. The alga was then cleaned and washed with sea water to remove epiphytes, detritus and encrusting material, and the resulting algal material was freeze-dried (Scanvac Cool Safe, LaboGene, Lynge, Denmark). The dry algal material was ground and stored at −80 °C until further use.

### 3.3. Extraction and Fractionation of Algal Extract by Vacuum Liquid Chromatography (VLC)

Freeze-dried samples of *S. coronopifolius* (470 g) were sequentially extracted in a 1:4 biomass:solvent ratio with MeOH and then CH_2_Cl_2_ at constant stirring for 12 h. Liquid-liquid extraction was additionally performed for the MeOH extract, using *n*-hexane to remove fats. The CH_2_Cl_2_ extract (5.2 g) was further concentrated and subjected to normal phase vacuum liquid chromatography on silica gel 60 (0.06–0.2 mm), using cyclohexane with increasing amounts (25%) of ethyl acetate (EtOAc) as the mobile phase (5 fractions, each one with 400 mL of eluent). The dried fractions were stored at −20 °C until further use (F1 287 mg, F2 412 mg, F3 796 mg, F4 442 mg, F5 159 mg).

### 3.4. Purification of Bromoditerpenes

Fraction F2 (412 mg) was further purified by preparative reversed-phase HPLC (XSelect CSH Phenyl-Hexyl 19 mm × 250 mm, 5 μm, Waters) at a flow rate of 12 mL/min and with a mixture of eluents H_2_O/CH_3_CN: isocratic from 0 to 5 min (25:75) and then a linear gradient from 5 to 20 min (from 25:75 to 15:85). This first purification afforded 6 subfractions, F2f1–F2f6, from which only compound **2** (F2p6, t_R_ 18.8 min, 23 mg) was pure. Fraction F2f1 (t_R_ 12.6 min, 80 mg) was subjected to a second purification step on a semi-preparative reversed-phase column (Synergi Fusion-RP 80 A, 10 mm × 250 mm, 4 μm, Phenomenex, Torrance, CA, USA) with an isocratic mode H_2_O/CH_3_CN (27:73) and a flow rate of 4.5 mL/min to afford pure compounds **3** (F2f1p1, t_R_ 17.5 min, 10 mg) and **5** (F2f1p2, t_R_ 19.0 min, 26 mg). Subfraction F2f3 (t_R_ 14.2 min, 9 mg) was purified on the same column with an isocratic mode H_2_O/CH_3_CN (20:80) at a flow rate of 5 mL/min to yield new compound **1** (F2f3p1, t_R_ 18.0 min, 4.5 mg).

The purification of fraction F3 (796 mg) was performed with the same procedure previously used for fraction F2, starting with a preparative reversed-phase HPLC separation. The elution was performed using mixtures of H_2_O/CH_3_CN under the following conditions: isocratic step from 0 to 5 min (30:70) and then a linear gradient until 25 min (from 30:70 to 15:85). This purification afforded pure compound **4** (F3p1, t_R_ 13.4 min, 19 mg), along with the four compounds previously purified from fraction F2.

### 3.5. Sphaerodactylomelol (**1**)

Colorless oil; [α]_D_^20^ −33.3 (*c* 0.15, CHCl_3_); UV (DAD) λ_max_ 255 nm; ECD (*c* 2.68 × 10^−4^ M, CH_2_Cl_2_) λ_max_ (Δε) 228 (0.95), 277 (−0.13) nm; IR (neat) σ 2968, 2927, 2855, 1457, 1437, 1377 cm^−1^; ^1^H and ^13^C NMR data, see Table 3; HRESIMS *m/z* 351.17117 and 353.16891 [M – OH]^+^ (50:50, calcd. for C_20_H_32_Br^+^, 351.16819 and 353.16614, Δ −8.5 and −7.8 ppm).

### 3.6. Biological Activities

#### 3.6.1. Cytotoxicity and Anti-Proliferative Activities

The anti-proliferative activities were performed on an *in vitro* carcinoma model of a human hepatocellular cancer (HepG-2), acquired in the American Type Culture Collection (ATCC). HepG-2 cells were cultured in RPMI 1640 (Sigma, Saint-Louis, MO, USA) medium supplemented with 10% fetal bovine serum (FBS) (Gibco, Grand Island, NY, USA) supplemented with 100 U/mL penicillin G, 0.25 µg/mL amphotericin B and 100 µg/mL streptomycin (Sigma, USA). The cells medium was changed every 3 days, and the cells reached confluence after 5–6 days of initial seeding in plates of a 25-cm^2^ growth area at a concentration of 42.2 × 10^6^ cells/ plate. For the subculture, cells were dissociated with trypsin-EDTA (Sigma, Saint-Louis, MO, USA), split into a 1:3 ratio and subcultured in Petri dishes with a 25-cm^2^ growth area. Cells were maintained in controlled conditions: 95% of humidified atmosphere, 5% of CO_2_ and 37 °C. Cells were seeded in 96-well plates, at a concentration of 4.4 × 10^4^ cells/well, for the cytotoxicity and anti-proliferative assays.

Cytotoxicity was evaluated after the cells reached total confluence and anti-proliferative activity after 36 h of initial seeding. Cells were incubated with crude extracts, VLC fractions and purified compounds, previously sterile filtered (0.2 µm, Whatman, Little Chalfont, UK), during 24 h at 1 mg/mL. For the samples with the highest activity, dose-response assays were done (10–1000 µg/mL; 24 h). Cisplatin (Sigma, St. Loiu, MO, USA) and tamoxifen (Sigma, Shanghai, China) were used as positive controls. The effects were estimated by the colorimetric assay based on the conversion of tetrazolium dye (MTT) (Sigma, Seelze, Germany) into a blue formazan product by living mitochondria [44]. After the treatment with bromoterpenes compounds, the cells medium was removed, and cells were washed with Hank’s medium (medium composition, in mM: NaCl 137, KCl 5, MgSO_4_ 0.8, Na_2_HPO_4_ 0.33, KH_2_PO_4_ 0.44, CaCl_2_ 0.25, MgCl_2_ 1.0, Tris HCl 0.15 and sodium butyrate 1.0, pH = 7.4). Cells were then incubated with MTT (1.2 mM), previously dissolved in Hank’s medium, during 4 h at 37 °C. The formazan products were dissolved in isopropanol (Panreac, Barcelona, Spain), contained 0.04 N HCl and were determined by the absorbance at 570 nm.

Results were expressed as IC_50_, defined as the concentration causing a 50% reduction or inhibition of cell viability and cell proliferation, respectively. 

#### 3.6.2. Antimicrobial Activities

Antimicrobial activities were evaluated by the capacity to inhibit *E. coli* (ATCC 25922), *P. aeruginosa* (ATCC 27853), *S. aureus* (ATCC 25923) and *C. albicans* (ATCC 10231) growth (OD 600_nm_). The crude extracts, VLC fractions, purified compounds and positives controls (ampicillin, bacitracin, chloramphenicol, oxytetracycline, amphotericin b and flumequine, from Sigma Aldrich, Canada) were prepared with sterile-filtered dimethylsulfoxide and stored at −20 °C. Tests were performed in 96-well plates at 37 °C for bacteria and 30 °C for fungi. Antimicrobial activity was expressed as IC_50_, defined as the concentration causing a 50% reduction of microorganism growth. 

#### 3.6.3. Data Analysis

IC_50_ were calculated from the analysis of non-linear regression using GraphPad Prism program even Y = 100/(1 + 10 (X − Log IC_50_)) equation.

## 4. Conclusions

In the present study, the chemical characterization of the major brominated diterpenes isolated from samples of *S. coronopifolius* collected in the Atlantic revealed five major compounds, including a new brominated dactylomelane diterpene, named sphaerodactylomelol (**1**), and four already known sphaerane bromoditerpenes, **2**–**5**. These molecules exhibited interesting high antimicrobial and antitumor activities against different targets and open new therapeutic potential. To the best of our knowledge, this is the first study on the isolation, identification and bioactive screening of bromoditerpenes isolated from *S. coronopifolius* collected from the Atlantic. Nonetheless, future studies will deeply describe the antitumor and antimicrobial activities and the mechanisms of action of the isolated compounds.

## Supplementary Files

Supplementary File 1
