# Supplementary material for: Antitumor and Antimicrobial Potential of Bromoditerpenes Isolated from the Red Alga, Sphaerococcus coronopifolius
Source: Mar Drugs. 2015 Jan 26;13(2):713–26. doi: 10.3390/md13020713 (PMC4344597; doi:10.3390/md13020713)
Supplement: Supplementary File 1 [file marinedrugs-13-00713-s001.pdf]

Supplementary Information

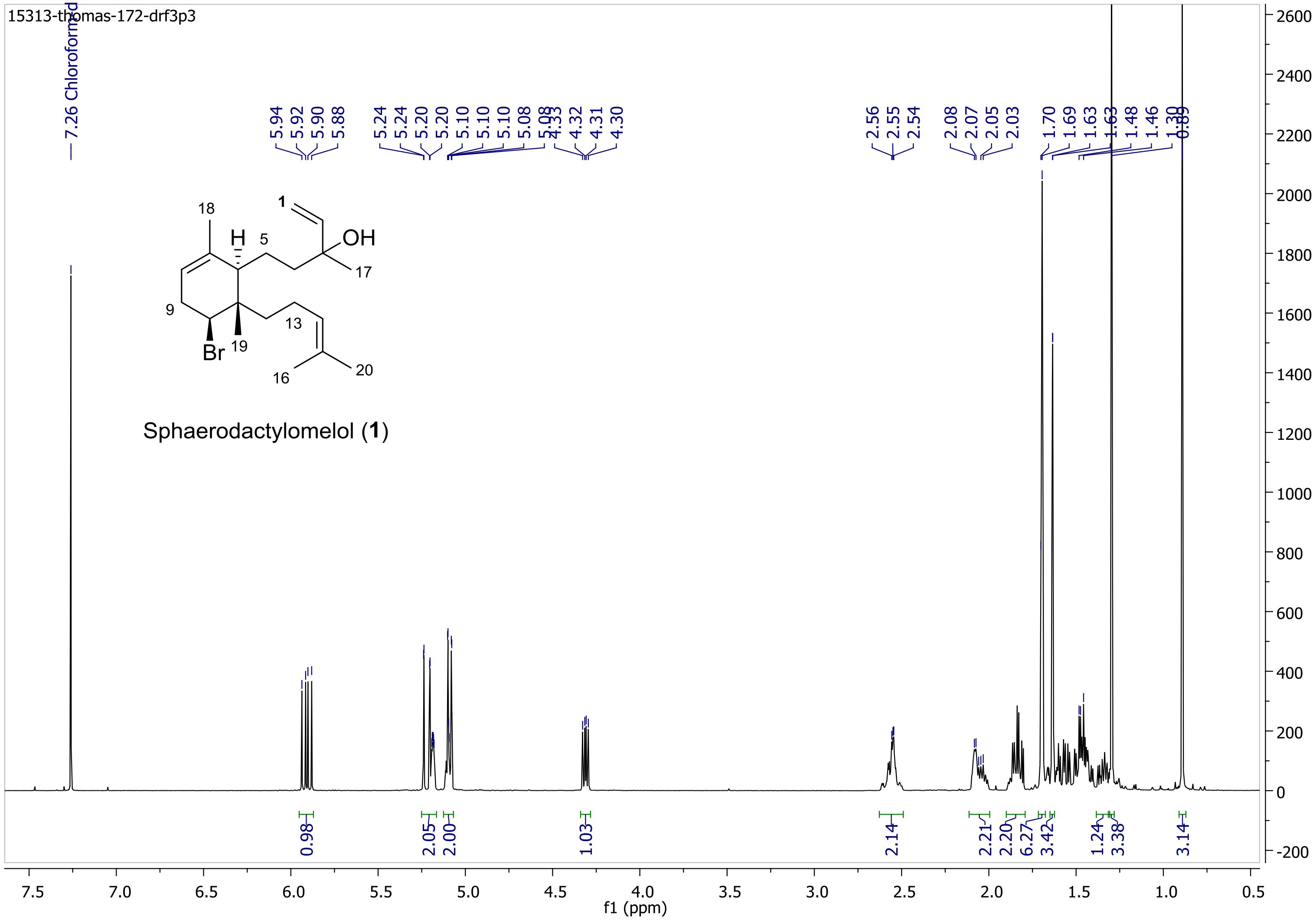

Figure S1. ^{1}H NMR spectrum of **1** (500 MHz) in $CDCl_3$.

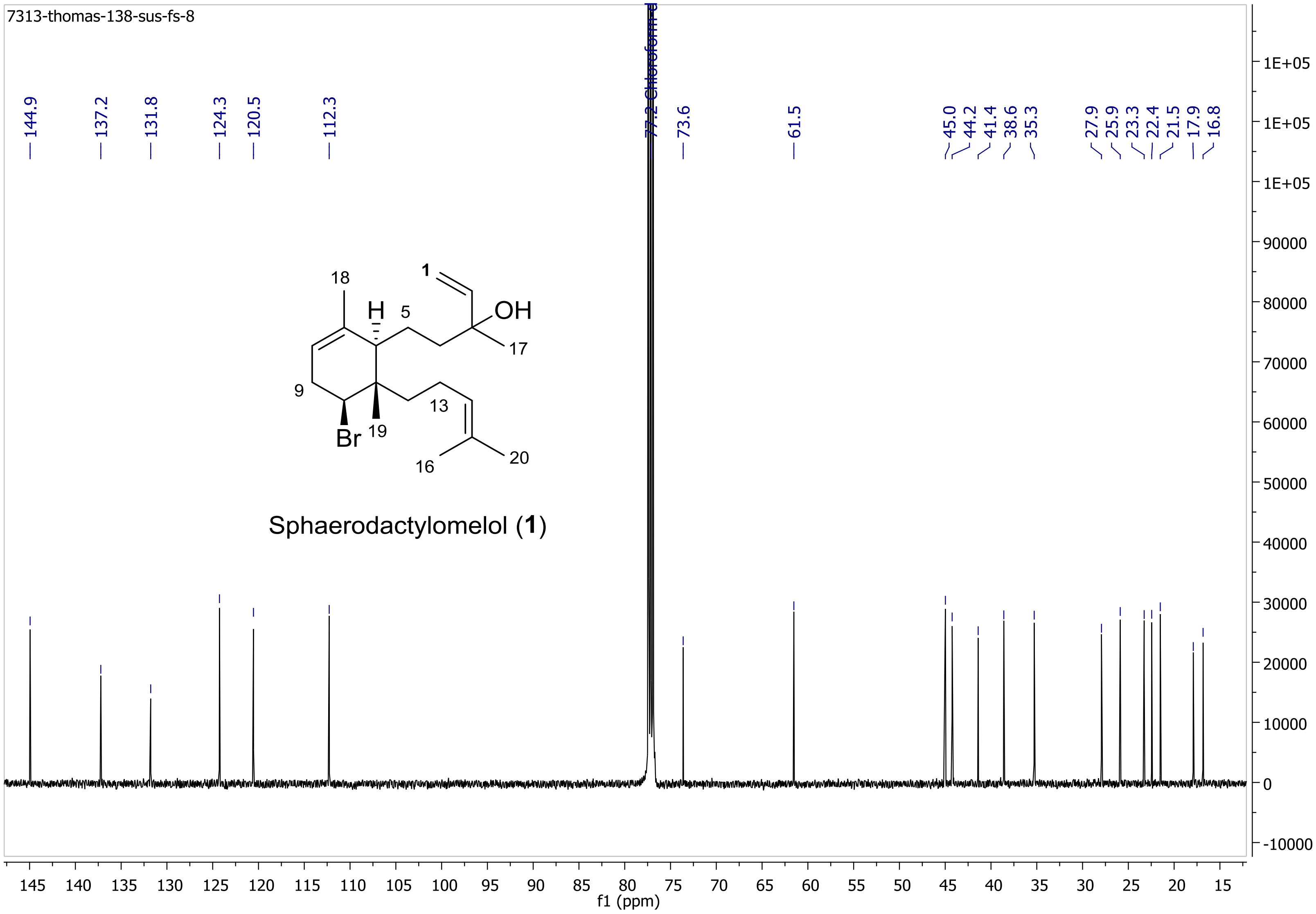

Figure S2. ^{13}C NMR spectrum of **1** (125 MHz) in CD_3OD.

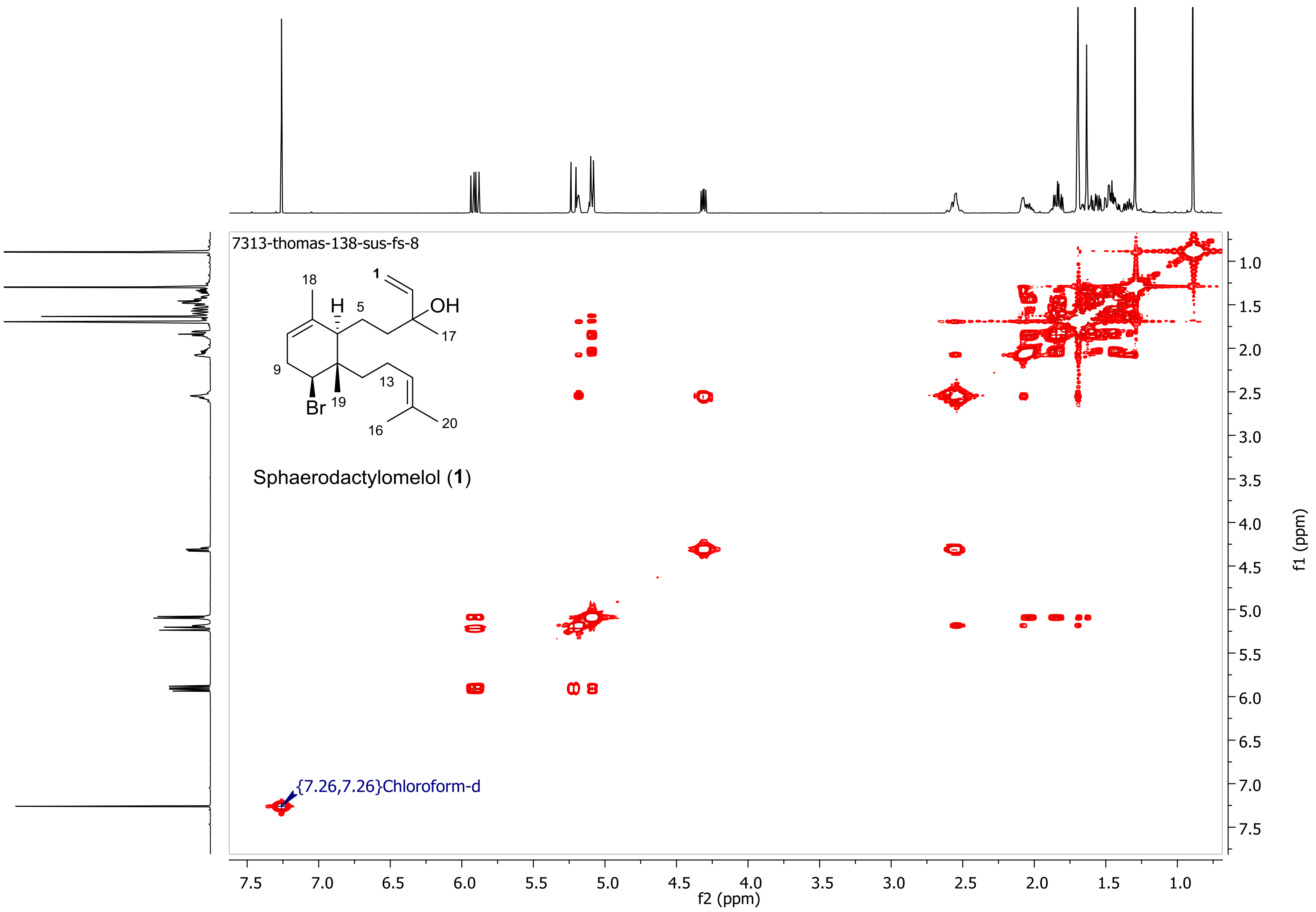

Figure S3. COSY spectrum of **1** in $CDCl_3$.

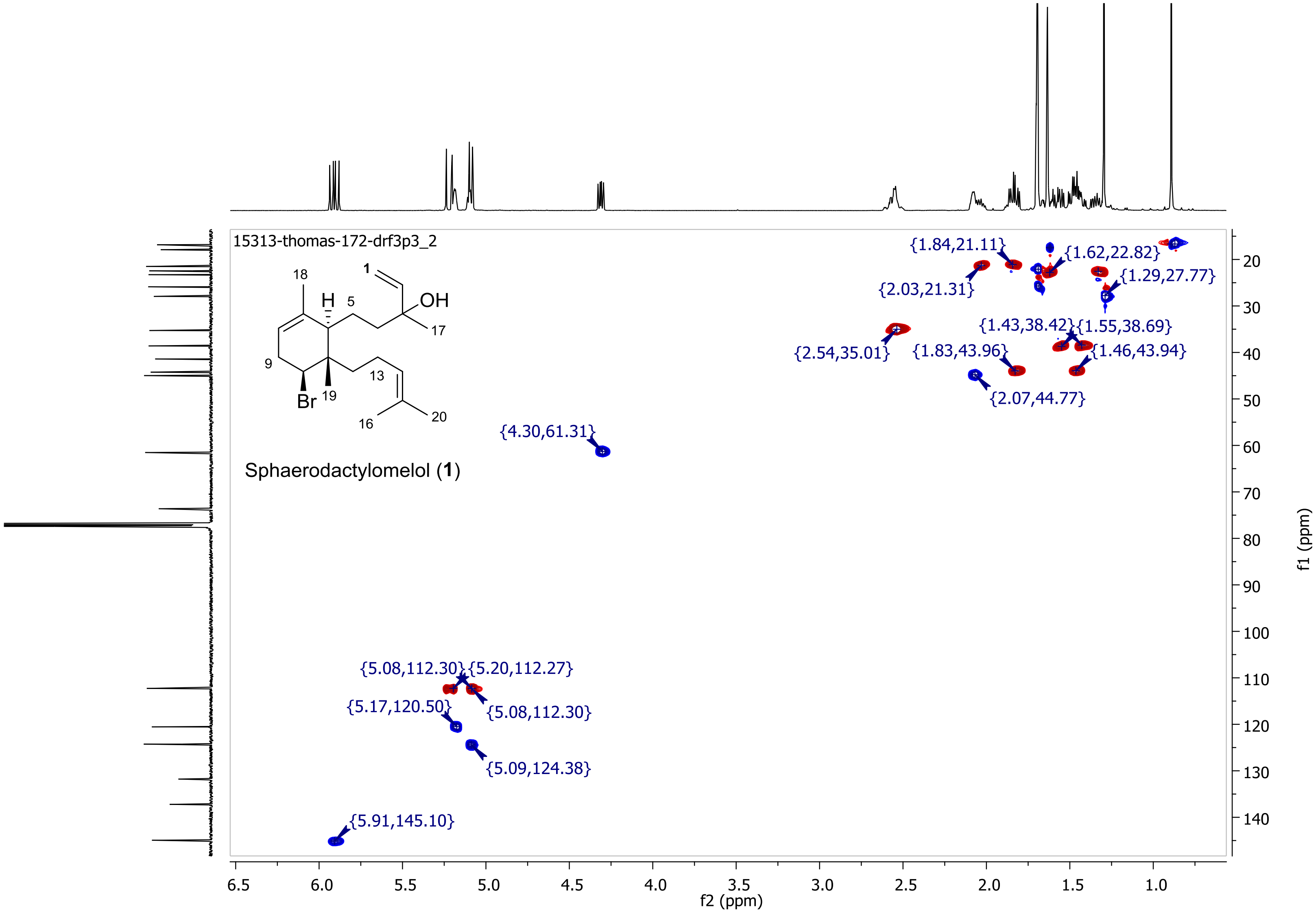

Figure S4. HSQC spectrum of **1** in $CDCl_3$.

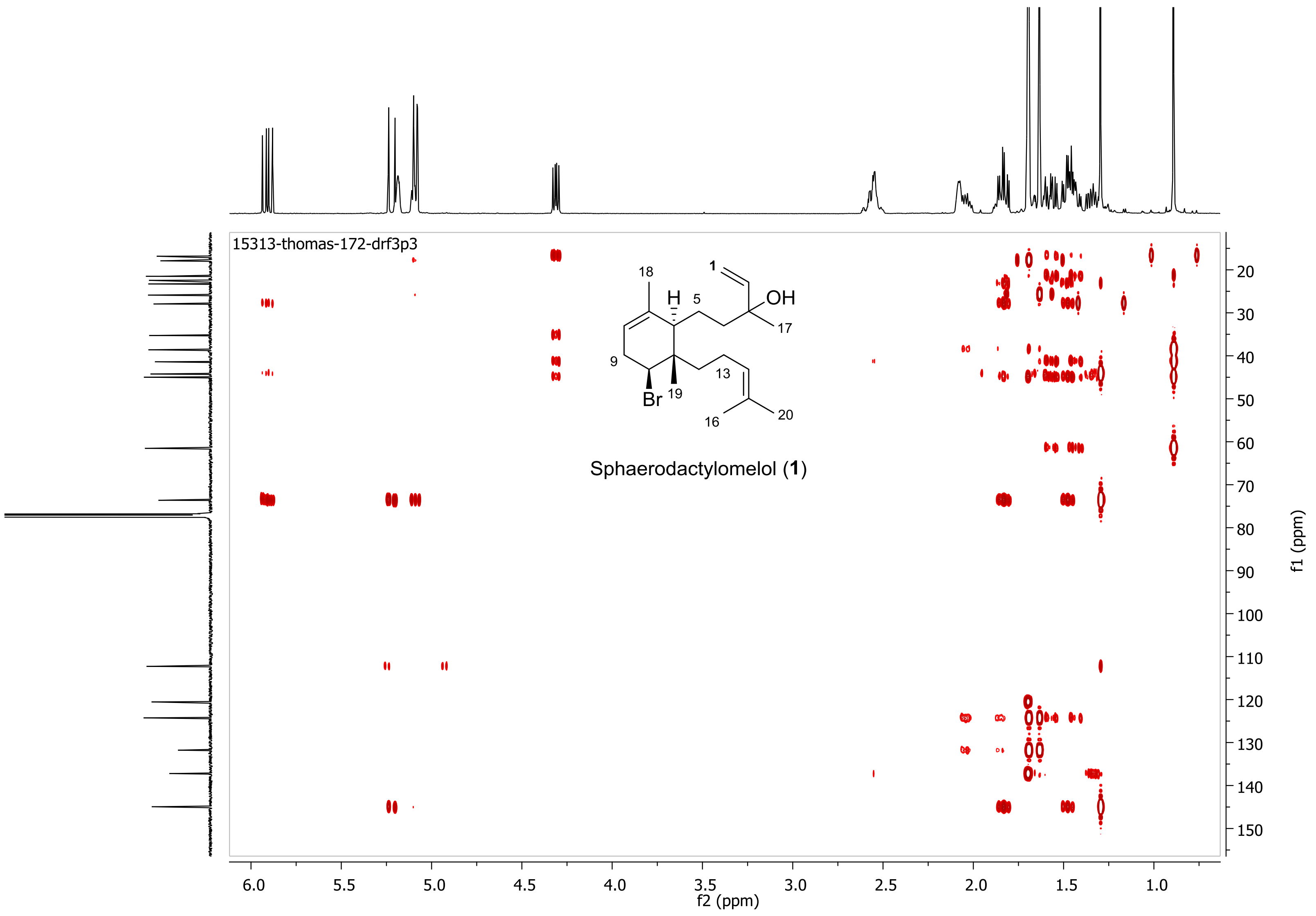

Figure S5. HMBC spectrum of **1** in $CDCl_3$.

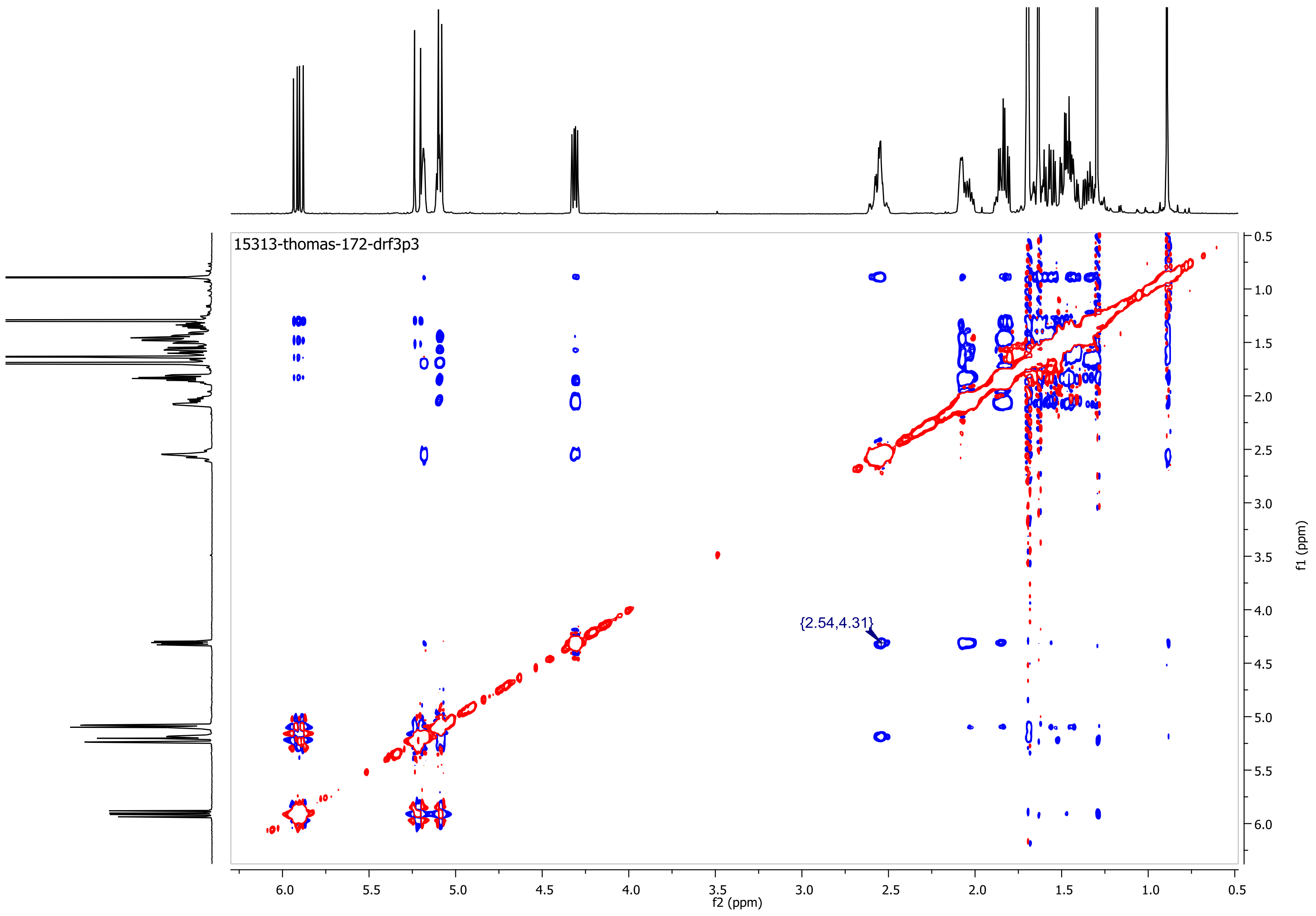

Figure S6. NOESY spectrum of **1** in $CDCl_3$.

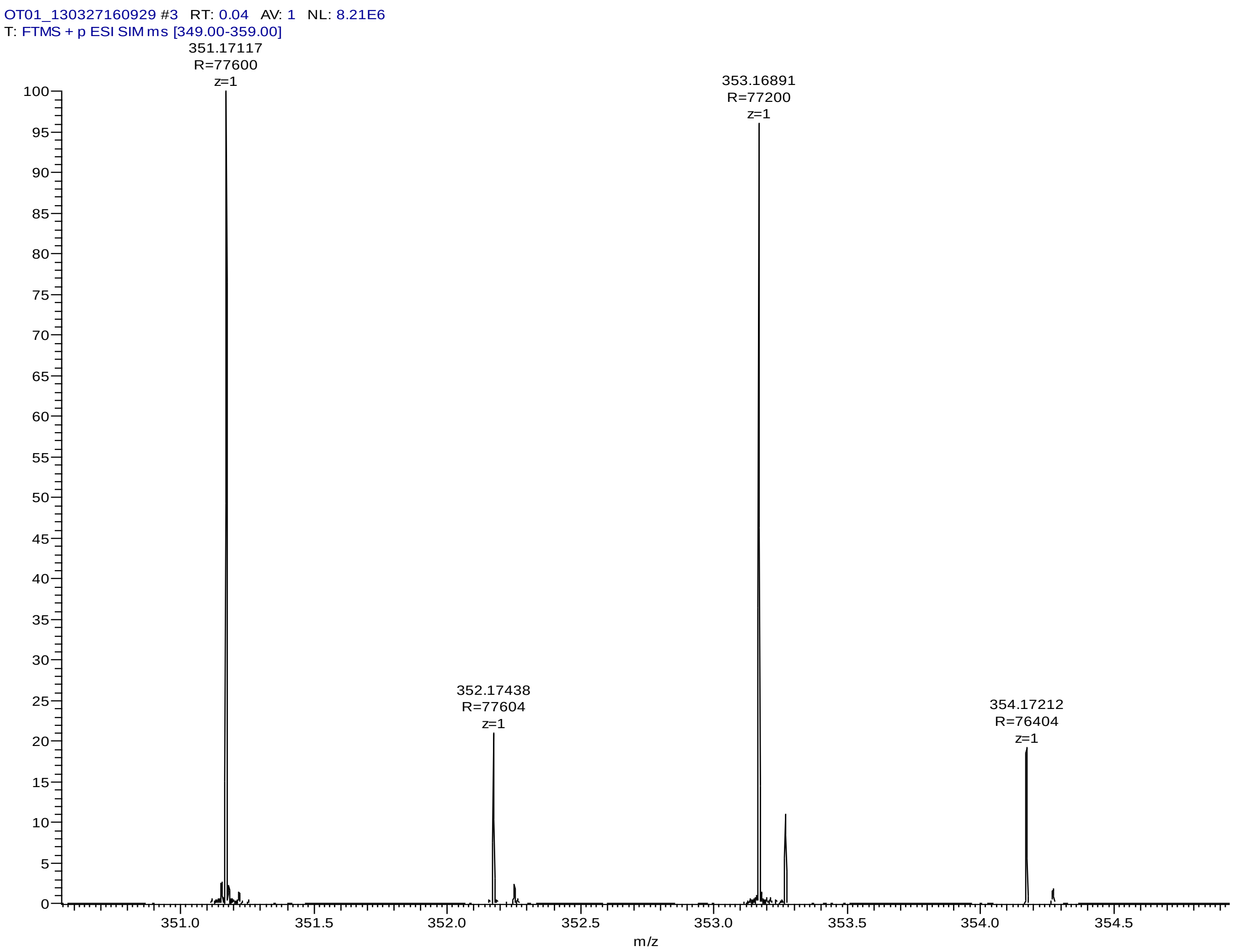

Figure S7. HRESIMS spectrum of **1**.

© 2015 by the authors; licensee MDPI, Basel, Switzerland. This article is an open access article distributed under the terms and conditions of the Creative Commons Attribution license (http://creativecommons.org/licenses/by/4.0/).
